# Reducing economic burden through split-shared care model for people living with uncontrolled type 2 diabetes and polypharmacy: a multi-center randomized controlled trial

**DOI:** 10.1186/s12913-024-11199-2

**Published:** 2024-06-22

**Authors:** Zheng Kang Lum, Jia Yeong Tan, Cynthia Sze Mun Wong, Zi Yin Kok, Sing Cheer Kwek, Keith Yu Kei Tsou, Paul John Gallagher, Joyce Yu-Chia Lee

**Affiliations:** 1 Department of Pharmacy and Pharmaceutical Sciences, Faculty of Science, MD1, Tahir Foundation Building, National University of Singapore, 12 Science Drive #06-03, Singapore, 117549 Singapore; 2 Keat Hong Family Medicine Clinic, Trilink Healthcare Private Limited, 2 Choa Chu Kang Loop, Singapore, #03-02 Singapore; 3 Bukit Batok Polyclinic, National University Polyclinics, 50 Bukit Batok West Ave 3, Singapore, 659164 Singapore; 4 Department of Clinical Pharmacy Practice, School of Pharmacy and Pharmaceutical Sciences, University of California, 101 Theory, Suite 100, Irvine, CA 92697 USA

**Keywords:** Collaborative care, Community pharmacist, Economic cost, Productivity loss, Split-shared care

## Abstract

**Background:**

Interprofessional collaborative care such as a split-shared care model involving family physicians and community pharmacists can reduce the economic burden of diabetes management. This study aimed to evaluate the economic outcome of a split-shared care model between family physicians and community pharmacists within a pharmacy chain in managing people with uncontrolled type 2 diabetes and polypharmacy.

**Method:**

This was a multi-center, parallel arm, open label, randomized controlled trial comparing the direct and indirect economic outcomes of people who received collaborative care involving community pharmacists (intervention) versus those who received usual care without community pharmacist involvement (control). People with uncontrolled type 2 diabetes, defined as HbA1c > 7.0% and taking ≥ 5 chronic medications were included while people with missing baseline economic data (such as consultation costs, medication costs) were excluded. Direct medical costs were extracted from the institution’s financial database while indirect costs were calculated from self-reported gross income and productivity loss, using Work Productivity Activity Impairment Global Health questionnaire. Separate generalized linear models with log link function and gamma distribution were used to analyze changes in direct and indirect medical costs.

**Results:**

A total of 175 patients (intervention = 70, control = 105) completed the trial and were included for analysis. The mean age of the participants was 66.9 (9.2) years, with majority being male and Chinese. The direct medical costs were significantly lower in the intervention than the control group over 6 months (intervention: -US$70.51, control: -US$47.66, p < 0.001). Medication cost was the main driver in both groups. There were no significant changes in productivity loss and indirect costs in both groups.

**Conclusion:**

Implementation of split-shared visits with frontline community partners may reduce economic burden for patient with uncontrolled type 2 diabetes and polypharmacy.

**Trial registration:**

Clinicaltrials.gov Reference Number: NCT03531944 (Date of registration: June 6, 2018).

**Supplementary Information:**

The online version contains supplementary material available at 10.1186/s12913-024-11199-2.

## Introduction

Diabetes imposes significant cost burden on global healthcare systems with direct economic cost of US$760 billion in diabetes spending projected to increase to US$845 billion by 2045 wordwide [1]. Studies have shown that diabetes-related complications, specifically coronary heart disease and myocardial infarction contributed to approximately 47.2% of all cause of deaths and almost twice the cost compared to those with diabetes but did not develop cardiovascular complications [2, 3]. While diabetes-related direct costs are taking a toll on the health economy at a global scale, indirect costs related to diabetes care also contribute to significant financial difficulties to the society and individuals. According to a large-scale national database study conducted in the United States, indirect cost attributed to 27.5% of the estimated annual health expenditures of US$327 billion for diabetes management [4]. Reduced labor force participation was the main indirect cost driver (42.8%), followed by presenteeism (30.7%), diabetes-associated pre-mature mortality (22.7%), and absenteeism (3.8%) [4]. Presenteeism is defined as reduced efficiency at work, while absenteeism is defined as absence from work due to health problems.

Antidiabetic medications are also the major drivers of diabetes cost burden. Diabetes is a condition without cure, hence in addition to lifestyle modification, people must rely heavily and chronically on pharmacotherapeutics to achieve and maintain control. A meta-analysis of 16 studies conducted in Germany between 2012 and 2018 concluded that medication cost accounted for approximately 50% of the total direct medical cost [5]. Another meta-analysis of 7 studies conducted among Eastern Mediterranean region countries also found medication cost incurred per patient to be the main cost driver of the total direct medical cost [6]. Poor adherence and medication-related adverse events and interactions have shown to further result in increased healthcare utilization and hospital admissions, feeding to the vicious cycle of diabetes-related economic burden [7].

A shared or split visit is defined as a visit in which a physician and other qualified health care professional(s) both provide the face-to-face and non-face-to-face work related to the visit [8]. The benefit of split-shared model is that it allows interprofessional collaboration and for the care providers to manage patients with expanded expertise and care individualization [8]. Numerous studies have shown the success of pharmacist-physician partnership adding value to diabetes outcomes, self-efficacy and medication safety [9]. This care model, however, is usually confined to healthcare providers and clinical pharmacists located within the same facility, failing to address convenience in care delivery and patient’s time lost in receiving the care in the clinic setting [10]. Given the fact that most people living with diabetes are community-based, interprofessional partnership with geographically advantageous frontline healthcare professionals such as the chain community pharmacists to manage diabetes is plausible. In this study, we conducted a randomized controlled trial to evaluate the economic impact of a collaborative split-shared care model involving community pharmacists and family physicians on diabetes management.

## Methods

### Study design and setting

This was a multi-center, parallel arm, open label, randomized controlled trial conducted in two primary healthcare institutions in Singapore over 6 months. Singapore’s prevalence of diabetes was 14.2% in 2020 and the economic cost of diabetes in this Southeast Asia country was projected to increase to US$1,867 million in 2050 [11, 12]. The clinical and humanistic findings, including details of the intervention, have been reported elsewhere [13]. The randomized controlled trial was registered with clinicaltrials.gov (NCT03531944). This study was approved by the National University of Singapore Institutional Review Board (reference number: H-18–019) and the National Healthcare Group Domain Specific Review Board (reference number: 2019/00201).

### Study participants

The inclusion criteria for the randomized controlled trial were age 21 years and above, diagnosed with type 2 diabetes mellitus, had baseline glycated hemoglobin (HbA1c) level of > 7.0%, and were taking 5 or more chronic medications (polypharmacy). Subjects who were mentally incapacitated or not able to communicate independently in the English, Chinese or Malay language were excluded. People with missing baseline economic data were also excluded.

### Study procedures

Upon signing of informed consent, direct medical costs of the study subjects were retrieved from the healthcare institutions’ electronic financial databases and indirect costs were derived from productivity loss which was assessed using a questionnaire administered at baseline and after 6 months. Demographic, medical and medication related data were extracted from the electronic medical records. The participants were randomized through stratified randomization based on baseline HbA1c (7.1 to 8.0%, 8.1 to 10.0%, ≥ 10.1%) before being sequentially allocated to the intervention or control group via flipping of a coin [13].

### Trial intervention

The trial intervention consisted of two face-to-face consultations and three telephonic follow-up sessions with the community pharmacist and “as scheduled” visits to the family physician [13]. The community pharmacists were engaged from a pharmacy chain store in Singapore. The first community pharmacist face-to-face consultation focused on medication therapy management and goal setting [13]. The second face-to-face consultation with the community pharmacist focused on self-monitoring of blood glucose (SMBG) education and interpretation. During the face-to-face consultations, the community pharmacist developed the medication action plan, including the address of any drug-related problems, together with the physician and patient. Each consultation on average lasted around 20 minutes. The community pharmacist subsequently followed up with the participants through telephonic consultations, focusing on lifestyle modifications, self-efficacy empowerment and motivational support [13]. The community pharmacists communicated with family physicians regularly to ensure seamless care.

### Outcome measures

#### Direct medical costs

The direct medical costs comprised of doctor consultation, care manager (nurse) consultation, pharmacist consultation, dietitian consultation, diabetic retinopathy screening, diabetic foot screening, medications, and laboratory tests. The direct medical costs were computed based on the gross costs in Singapore Dollars (SGD) and converted to the USD based on the currency conversion rate on March 31, 2020 (SGD 1 = US$0.70364). Direct medical costs were derived from the gross charges of the clinic for the respective services, and these charges were accurate as of 2020.

The total consultation cost was the sum of the doctor, care manager, pharmacist and other allied health professional consultations, diabetic retinopathy screening, and diabetic foot screening (Table 1). Medication costs were separated into diabetes medications and non-diabetes medications. Changes in direct medical costs over 3 time-points were analyzed: (1) baseline, (2) 3-month, and (3) 6-month. Baseline refers to the first physician consultation and as needed same-day diabetic retinopathy or foot examination, and consultations for lifestyle or diet modifications conducted by nurse or dietitian. At the 3-month time point, it included the second physician consultation and as needed same-day diabetic retinopathy or foot examination, and consultations for lifestyle or diet modifications conducted by nurse or dietitian, as well as pharmacist consultation on glucose monitoring and goal setting. The activities at 6-month were similar to 3-month, including follow-up on glycemic goals.

**Table 1:** Details on Cost Variables

Direct Medical Costs^a^	Unit Price (SGD)	Unit Price (USD)
Doctor consultation	50.00	35.18
Care manager (nurse) consultation	48.14	33.87
Pharmacist consultation	7.00	4.93
Diabetic foot screening	35.00	24.63
Diabetic retinopathy screening	40.00	28.15

*Abbreviations: SGD* Singapore Dollars, *USD* United States Dollars

^a^Based on the clinic gross charges in 2020

#### Indirect costs

The indirect costs assessed in this study were incurred from productivity losses. The participants’ productivity losses (comprising of work productivity and activity impairment) were assessed using the 6-item valid and reliable Work Productivity Activity Impairment – Global Health (WPAI-GH) questionnaire (appended in the Supplementary File) [14].

In order to assess work productivity loss and activity impairment, scores were calculated based on the four domains: (1) absenteeism, (2) presenteeism, (3) work impairment, and (4) activity impairment [14]. Absenteeism were computed based on number of hours missed from work due to health problems, adjusted for the number of work hours missed due to non-health problems and actual number of work hours [14]. Presenteeism was computed based on the score of item 5 [14].

Indirect costs attributed to absenteeism and presenteeism were calculated using the participants’ gross monthly income in Singapore Dollars and the total number of hours worked per month. Absenteeism, in percentage, was multiplied by the total number of working hours for the past 7 days to derive the number of hours missed from work due to health-related reasons. This was then approximated to the number of hours missed per month using the expression, number of hours missed from work due to health-related reasons in the past 7 days divided by 7 and multiply by 30 [15]. The number of hours missed from work due to health-related reasons per month was multiplied by the hourly wage rates to derive the indirect cost incurred due to absenteeism in a month [15].

The indirect cost attributed to presenteeism, which was defined as the number of hours of work affected by health-related problems, was also calculated as per the calculation for indirect cost incurred due to absenteeism. The total indirect cost was the sum of the indirect costs due to absenteeism and presenteeism. The indirect costs computed in Singapore Dollars (SGD) were converted to the USD based on the currency conversion rate on March 31, 2020 (SGD 1 = USD 0.70364).

### Data analysis

Descriptive analysis of the baseline characteristics of the participants were performed. The baseline characteristics between both groups were compared using Student’s t test or Mann Whitney U test and chi square test, as appropriate.

Separate generalized linear models (GLM) with the log link function and gamma distribution were used to analyze the impact of the community pharmacist-involved collaborative split-shared care model on total direct medical cost and its component costs per participant. The models included covariates such as duration of diagnosed diabetes, total number of comorbidities, and smoking status.

Changes in absenteeism, presenteeism, work impairment, and activity impairment over 6 months were analyzed using the non-parametric Mann Whitney U test. Another GLM with the log link function and gamma distribution was used to analyze the impact of the community pharmacist-involved collaborative care model on indirect medical costs incurred per participant. The GLM also adjusted for covariates such as duration of diagnosed diabetes, total number of comorbidities, and smoking status. Single imputation was used for handling missing data.

Sample size for the randomized controlled trial was calculated using an estimated mean HbA1c change between intervention and control arms over 6 months of 0.44% [16]. With a standard deviation of 1%, type I error rate of 5%, and power of 80%, the calculated total sample size was 164 participants. Per protocol analysis was conducted. A p value of less than 0.05 was considered statistically significant in all analyses. The Statistical Package for Social Sciences (V26.0; SPSS Inc, Chicago, Illinois) was used for all computations in this study.

## Results

### Participant demographics

A total of 264 subjects were recruited in the randomized controlled trial, with 89 participants (Intervention (INT): 61 [68.5%] vs. Control (CTL): 28 [31.5%]) dropped out and left with 175 participants for the economic analysis (INT: 70 vs. CTL: 105). Reasons for dropout included general disinterest or uncontactable (INT: 7 and CTL: 10), no longer receiving care at trial sites (INT: 11 and CTL: 11), unwillingness to perform SMBG (INT: 7 and CTL: 0), not keen on survey participation (INT: 0 and CTL: 5), not keen on pharmacist intervention (INT: 36 and CTL: 0), and deaths (*n* = 2 [2.2%]; INT: 0 and CTL: 2).

The mean age of the participants was 66.9 (SD 9.2) years (INT: 68.0 (SD 8.3) vs. CTL: 66.2 (SD 9.8)), majority of them were males (INT: 35 [50.0%] vs. CTL: 59 [56.2%]) and Chinese (INT: 46 [65.7%] vs. CTL: 81 [77.1%]). Majority of the participants had at least high school education (INT: 40 [57.2%] vs. CTL: 59 [56.1%]) and were married (INT: 53 [75.7%] vs. CTL: 87 [82.9%]). Out of the 70 participants in the intervention group, 30 (42.9%) of them were employed. For the control group, 50 (47.6%) out of 105 participants were employed.

The baseline mean HbA1c level of the participants was approximately 8% and the participants had on average 5 comorbidities and were taking on average approximately 7 chronic medications. In terms of diabetes regimen, most of the participants were on only oral antidiabetic agents (INT: 53 (75.7%) vs. CTL: 74 (70.5%)). The baseline characteristics between the intervention and control groups were comparable (Table 2).

**Table 2:** Baseline characteristics of trial participants

Characteristics	Intervention Group (*n* = 70)	Control Group (*n* = 105)	*p*-value
Age, years	68.0 ± 8.3	66.2 ± 9.8	0.216
Gender			0.421
Male	35 (50.0)	59 (56.2)	
Female	35 (50.0)	46 (43.8)	
Ethnicity			0.089
Chinese	46 (65.7)	81 (77.1)	
Malay	11 (15.7)	17 (16.2)	
Indian	12 (17.1)	7 (6.7)	
Others	1 (1.4)	0 (0.0)	
Educational status			0.851
No formal education	7 (10.0)	15 (14.3)	
Elementary	23 (32.9)	31 (29.5)	
High school	27 (38.6)	39 (37.1)	
College / University	13 (18.6)	20 (19.0)	
Marital status			0.472
Single	4 (5.7)	5 (4.8)	
Married	53 (75.7)	87 (82.9)	
Separated / divorced / widowed	13 (18.6)	13 (12.4)	
Employment status			0.536
Unemployed	40 (57.1)	55 (52.4)	
Employed	30 (42.9)	50 (47.6)	
HbA1c, %	8.1 ± 1.1	8.2 ± 0.9	0.516
Number of comorbidities	5.3 ± 1.6	5.1 ± 1.4	0.317
Duration of diabetes, years	13.9 ± 9.8	14.8 ± 9.6	0.522
Number of chronic medications	6.9 ± 2.0	7.0 ± 1.8	0.803
Diabetes regimen			0.328
Insulin only	0 (0.0)	3 (2.9)	
Oral hypoglycemic agents only	53 (75.7)	74 (70.5)	
Insulin and oral hypoglycemic agents	17 (24.3)	28 (26.7)	

All values were reported in mean ± standard deviation or number (percentage) as appropriate

*Abbreviations: HbA1c* Glycated hemoglobin

### Direct medical costs

The total direct medical cost incurred per participant in the intervention group at baseline was US$162.33 (SD 92.25) which decreased significantly to US$138.17 (SD 92.89) at 3-month and further decreased to US$91.82 (SD 76.51) at 6-month (*p* < 0.001) (Table 3). Among the participants in the control group, the total direct medical cost incurred per participant significantly decreased from US$167.06 (SD 94.17) at baseline to US$158.68 (SD 129.19) at 3-month to US$119.40 (SD 105.19) at 6-month (*p* = 0.004) (Table 3). The decrease in total direct medical cost incurred per participant over the 6 months was significantly greater in the intervention group as compared to the control group (INT: US$70.51 vs. CTL: US$47.66, *p* = 0.044).

**Table 3:** Direct medical costs incurred per participant over 6 months (*N* = 175)

**Cost Component**^a^	**Intervention (*n* = 70)**				**Control (*n* = 105)**			
	**Baseline**	**3-Month**	**6-Month**	***p*-value**^**‡**^	**Baseline**	**3-Month**	**6-Month**	***p*-value**^**‡**^
Consultation	63.63 (19.22)	58.78 (23.25)	56.80 (20.53)	0.145	37.90 (21.89)	35.72 (24.04)	33.25 (23.22)	0.344
Doctor	31.48 (8.55)	25.28 (14.76)	27.28 (13.04)	0.012^*^	29.47 (11.87)	28.16 (12.20)	25.66 (13.78)	0.087
Nurse	2.40 (8.08	3.91 (9.11)	0.78 (4.80)	0.054	2.60 (7.68)	1.90 (6.25)	1.80 (6.37)	0.650
DRP	1.67 (6.83)	0.90 (5.33)	2.08 (7.91)	0.582	2.17 (8.09)	1.03 (5.87)	2.41 (8.20)	0.363
DFS	3.46 (9.42)	4.06 (10.79)	2.02 (7.74)	0.420	3.67 (9.96)	4.62 (11.75)	3.39 (9.18)	0.665
Medication	101.51 (79.20)	86.41 (83.54)	48.65 (68.12)	< 0.001^*^	106.15 (84.21)	105.96 (120.08)	69.98 (89.46)	0.010^*^
Diabetes	51.82 (62.20)	47.82 (64.40)	20.74 (37.77)	0.002^*^	57.95 (66.49)	51.23 (67.44)	39.62 (63.25)	0.126
Non-diabetes	49.69 (38.32)	38.59 (41.49)	27.91 (47.31)	0.011^*^	48.20 (42.61)	54.73 (93.04)	30.36 (43.71)	0.018^*^
Laboratory	11.41 (15.16)	11.94 (14.60)	9.79 (14.32)	0.666	14.94 (16.90)	13.00 (15.42)	10.65 (15.66)	0.152
Total Cost	162.33 (92.25)	138.17 (92.89)	91.82 (76.51)	< 0.001^*^	167.06 (94.17)	158.68 (129.19)	119.40 (105.19)	0.004^*^

^**‡**^Denotes changes in costs over 6 months within each group

^*^Denotes statistically significant changes in costs over 6 months

^a^All cost data are presented in mean (standard deviation). All costs were converted from Singapore Dollars to United States Dollars as per the currency conversion rate on March 31, 2020 (SGD 1 = USD 0.70364)

In terms of the component costs (consultation, medication, and laboratory), the decrease in total medication cost incurred per participant over 6 months was significantly greater in the intervention group as compared to the control group (between-group difference: US$16.69, *p* = 0.039). The decrease in total medication cost per participant within each group over 6 months was also significant (INT: US$52.86, *p* < 0.001 vs. CTL: US$36.17, *p* = 0.010). The decrease in total consultation cost per participant over 6 months was not significantly different between the intervention and control groups (INT: US$6.83 vs. CTL: US$4.65, *p* = 0.746). Similarly, the change in laboratory cost incurred per participant was not significantly different between the two groups (INT: US$1.62 vs. CTL: US$4.29, *p* = 0.901).

At baseline, the main cost driver for each participant in the intervention group was total medication cost, accounting for approximately 62.5% of their total direct medical cost. Medication cost remains the main cost driver for this group of participants after 3 months (62.5% of their total direct medical cost). At 6-month, while medication cost remains the main cost driver, it accounted for a lower percentage of the total direct medical cost incurred per participant in the intervention group (53.0%).

Within the control group, medication cost was also the main cost driver at baseline, accounting for approximately 63.5% of the total direct medical cost. At 3-month, medication cost, being the main cost driver, accounted for 66.8% of the total direct medical cost, and at 6-month, medication cost accounted for 58.6% of the total direct medical cost for the participants in the control group.

### Indirect costs

The baseline absenteeism among participants in the intervention and control groups were low at 0.8% and 1.8% respectively (*p* = 0.323) (Table 4). There was minimal and insignificant change in absenteeism among participants in the intervention group (change: -0.01%, *p* = 0.984). There was an increase in absenteeism among participants in the control group (change: + 0.60%, *p* = 0.602). Similarly, the changes over 6 months in presenteeism, work impairment, and activity impairment within each group were also not significantly different (Table 4). In both groups, presenteeism was a greater contributor to work impairment than absenteeism over the 6-month period.

**Table 4:** Productivity loss and indirect costs incurred per participant over 6 months (*N* = 175)

**Productivity, %**^a^	**Intervention (*n* = 70)**			**Control (*n* = 105)**		
	**Baseline**	**6-Month**	***p*-value**^**‡**^	**Baseline**	**6-Month**	***p*-value**^**‡**^
Absenteeism	0.8 (3.0)	0.8 (2.8)	0.984	1.8 (6.0)	2.4 (6.6)	0.602
Presenteeism	8.3 (11.0)	7.8 (10.9)	0.868	18.3 (20.9)	19.8 (18.8)	0.725
Work impairment	8.9 (12.4)	8.5 (11.2)	0.905	19.3 (22.2)	21.2 (20.1)	0.650
Activity impairment	16.6 (22.9)	13.3 (14.5)	0.322	22.8 (27.6)	22.2 (22.6)	0.848
**Indirect Cost, USD**^b^						
Absenteeism cost	1.28 (6.43)	1.46 (6.80)	0.918	8.61 (32.22)	9.70 (23.71)	0.854
Presenteeism cost	19.48 (28.96)	22.61 (39.44)	0.737	67.73 (115.66)	72.14 (79.22)	0.852
Total indirect cost	20.76 (31.63)	24.08 (41.54)	0.739	76.34 (133.58)	81.85 (128.47)	0.840

^**‡**^Denotes changes in costs over 6 months within each group

^a^All data are presented in mean (standard deviation)

^b^All costs were converted from Singapore Dollars to United States Dollars as per the currency conversion rate on March 31, 2020 (SGD 1 = USD 0.70364)

There was a greater but insignificant increase in indirect costs over 6 months for participants in the intervention group as compared to the control group (INT: US$3.32, vs. CTL: US$5.51, *p* = 0.368) (Table 4). While there were no significant changes in the indirect costs for both intervention and control groups, we observed that presenteeism contributed approximately 90% of the total indirect costs.

## Discussion

With the shift in paradigm from a physician-centric care model to a person-centric collaborative care model that anchored on shared decision-making between healthcare providers and patients in managing diabetes, this study found superior economic evidence in the engagement of community pharmacist as part of a collaborative split-shared care model. The partnership between community pharmacist with family physician improves access to care for people living with diabetes in the community. Community pharmacists also bring convenience for people living with diabetes to access care readily, especially amongst those who are employed and often have to miss work to attend to follow-up appointments in the physician clinic [17]. The collaborative split-shared care model has led to a significant decrease in total direct medical cost. Furthermore, there were significant reductions in doctor consultation cost and medication cost among participants in the intervention group. There were no significant changes in productivity and indirect costs within the intervention and control groups. This study highlighted the positive impact of the collaborative split-shared care model on direct medical cost of people living with type 2 diabetes. This pharmacoeconomic analysis was pre-planned in the trial and this analysis was taken into account when designing the trial.

Diabetes is a complex and multi-faceted chronic disease that requires optimal pharmacotherapeutic management coupled with demanding self-management regimen. This translates to significant financial hardship on individuals, and it was found that people with diabetes incurred approximately 2 times higher in medical expenditures than they would in the absence of diabetes [18]. For example, among 17,391 adults with diabetes in the United States, 41.1% of them were part of families who had financial hardships from medical bills and of which 15.6% of these families were not able to pay medical bills at all [19]. Financial hardships sustained from medical expenses can result in significant distress (odds ratio (OR): 1.14, 95% CI: 1.05, 1.24), medication non-adherence (OR: 1.43, 95% CI: 1.30, 1.57), and foregone care (OR: 1.30, 95% CI: 1.20–1.40) [19]. These factors can in turn affect glycemic control negatively, leading to additional medications and eventually further increase in medical cost [20]. In addition, financial burden of diabetes was also established as a barrier to optimal self-management among participants from Europe, Australia, Asia, and America [21]. Therefore, the collaborative care model involving community healthcare providers can be a solution in reducing total direct medical costs and alleviating part of the financial hardships experienced by community dwelling patients with type 2 diabetes.

In our study, medication was found to be the main cost driver, contributing approximately 65% to total direct medical cost. This was also consistent with previous studies that reported medication as the main cost driver [5, 6, 22]. Notably, involving community pharmacist as part of the split-shared care model has led to a significantly greater reduction in medication cost. This positive outcome can be attributed to the targeted intervention and clinical activities performed by the community pharmacist which included individualized medication therapy management and lifestyle empowerment. The trial participants were taking on average 7 chronic medications, and involving community pharmacists can potentially address and simplify therapeutic regimen based on the values and preferences of the patient, while not compromising outcomes [23]. Given that regimen complexity is well-established as a barrier to medication adherence, simplifying regimen can also potentially improve adherence [24]. In addition to leveraging on the medication expertise of community pharmacist, this trial also involved active direct communication and decision-making between the pharmacist, physician, and patient. This form of collaboration has been found to significantly reduce medication cost and also improve overall glycemic control [16, 25, 26]. This type of collaborative care model has also found to reduce healthcare utilizations and hospitalizations among people living with diabetes and other chronic diseases, leading to an estimated annual cost saving of US$2,619 per patient [27].

People with diabetes not only incurred significant direct medical cost but also productivity loss and indirect costs. Productivity loss can impose economic burden on society, employers and individuals through the reduction in income earnings, tax revenue, and gross domestic product (GDP) [4]. For example, in Vietnam, the healthcare expenditure per person with diabetes has increased from $38 per capita to $217 per capita over 10 years from 2007 to 2017, with indirect costs accounting for 12% to 68% [28]. In the United States, presenteeism, defined as reduced efficiency at work, drove around 30% of the total indirect cost while absenteeism accounted for approximately 4% [4]. In China, a total of $2.6 trillion in lost GDP was attributed to reduced productivity among people with diabetes [29]. Multidisciplinary collaborative care model has been suggested as one of the potential care delivery models that can reduce indirect economic costs [30]. Our trial found that there were no significant reduction in productivity loss and indirect costs among participants in the collaborative care model. Productivity losses and indirect costs were estimated from gross monthly income, and hence this could be due to the already low baseline productivity loss and indirect costs as most of the employed participants were lower-wage workers with an overall mean monthly gross income at US$750. Furthermore, this implied that no additional indirect costs were incurred due to the intervention.

Some participants in the intervention group dropped out as they were not keen on the pharmacist’s services. This can potentially be a barrier in future implementation of this care model. Having doubts over the quality of care may be one of the key barriers to implementing the pharmacist’s services in the community [31]. Community outreach programmes should be organized to raise the awareness of the roles of pharmacists as part of an interprofessional care team. Future research may be required to elucidate facilitators and barriers to implementation of such care model.

This study had a few limitations. The direct medical costs evaluated only reflected expenses in the primary care setting and did not include inpatient costs that may have incurred to the study patients during the study period. However, this is still reflective of the direct medical cost incurred by the participants as the primary care clinic is the primary site of care that the participants followed up for their diabetes and chronic diseases. While the dropout rate was higher in the intervention group, it was comparable to other trials of similar nature and intervention. Subjects were recruited from government-owned clinics where all patients received subsidized medical care hence it is unlikely to contribute to drastic impact unlike studies where private clinics may be involved where their patients are more heterogeneous in nature. In addition, our analysis on the economic outcomes of those who dropped out in the intervention group were not significantly different from the control group. Future studies may want to consider elucidating perspectives of people who dropped out of these type of split-shared care models. While the dissemination of this manuscript was delayed by the pandemic, the findings remain relevant, especially as the global healthcare landscape shifts towards mixed care models. Future research should evaluate the impact of community pharmacist-involved collaborative care model beyond 6 months, and to include healthcare utilizations and other aspects of indirect cost such as pre-mature mortality. With the split-shared care model showing significant direct medical cost reduction, future studies may want to analyse specific characteristics or segments of the population who may experience greater economic benefits from this type of care model.

## Conclusion

This study found a significant reduction in total direct medical cost, medication cost, and physician consultation cost incurred among people who had diabetes managed through the collaborative split-shared care model. There was no additional increase in indirect costs or productivity loss. Involving community pharmacist as part of a collaborative split-shared care model can be a potential solution to reduce economic burden. Furthermore, this care model may bring convenience to people living with diabetes in the community, and consequentially improve access and adherence to care.

### Supplementary Information

Supplementary Material 1.

## Data Availability

The datasets generated and/or analysed during the current study are not publicly available due confidential information.

## References

[1] Williams R, Karuranga S, Malanda B (2020). Global and regional estimates and projections of diabetes-related health expenditure: Results from the International Diabetes Federation Diabetes Atlas, 9th edition. Diabetes Res Clin Pract.

[2] Bommer C, Sagalova V, Heesemann E (2018). Global economic burden of diabetes in adults: projections from 2015 to 2030. Diabetes Care.

[3] Einarson TR, Acs A, Ludwig C, Panton UH (2018). Economic burden of cardiovascular disease in type 2 diabetes: a systematic review. Value Health.

[4] American Diabetes Association (2018). Economic costs of diabetes in the U.S. in 2017. Diabetes Care.

[5] Stegbauer C, Falivena C, Moreno A (2020). Costs and its drivers for diabetes mellitus type 2 patients in France and Germany: a systematic review of economic studies. BMC Health Serv Res.

[6] Ansari-Moghaddam A, Setoodehzadeh F, Khammarnia M, Adineh HA (2020). Economic cost of diabetes in the Eastern Mediterranean region countries: A meta-analysis. Diabetes Metab Syndr.

[7] Formica D, Sultana J, Cutroneo PM (2018). The economic burden of preventable adverse drug reactions: a systematic review of observational studies. Expert Opin Drug Saf.

[8] Aimee L. W. Keeping up on Split or Shared Service Changes. Find-A-Code Articles. Published 2023. https://www.findacode.com/articles/keeping-up-split-shared-service-changes-37328.html. Accessed 25 Sept 2023.

[9] Abdulrhim S, Sankaralingam S, Ibrahim MIM, Awaisu A (2020). The impact of pharmacist care on diabetes outcomes in primary care settings: An umbrella review of published systematic reviews. Prim Care Diabetes.

[10] Ulrich IP, Patel S, Gilmer B (2019). Evaluation of a pharmacist-physician covisit model in a family medicine practice. J Am Pharm Assoc JAPhA.

[11] International Diabetes Federation. IDF Western Pacific members (Singapore). https://idf.org/our-network/regions-members/western-pacific/members/113-singapore.html. Accessed 28 Oct 2020.

[12] Png ME, Yoong J, Phan TP, Wee HL (2016). Current and future economic burden of diabetes among working-age adults in Asia: conservative estimates for Singapore from 2010–2050. BMC Public Health.

[13] Lum ZK, Chang KL, Tsou KYK (2022). Enhancing diabetes care with community pharmacist-involved collaborative care model: A multi-centre randomised controlled trial. Diabetes Res Clin Pract.

[14] Reilly MC, Zbrozek AS, Dukes EM (1993). The validity and reproducibility of a work productivity and activity impairment instrument. Pharmacoeconomics.

[15] Suwa K, Flores NM, Yoshikawa R, Goto R, Vietri J, Igarashi A (2017). Examining the association of smoking with work productivity and associated costs in Japan. J Med Econ.

[16] Siaw MYL, Lee JYC (2019). Multidisciplinary collaborative care in the management of patients with uncontrolled diabetes: A systematic review and meta-analysis. Int J Clin Pract.

[17] Peterson J, Hinds A, Garza A (2020). Impact of physician-pharmacist covisits at a primary care clinic in patients with uncontrolled diabetes. J Pharm Pract.

[18] Mszar R, Grandhi GR, Valero-Elizondo J (2020). Cumulative burden of financial hardship from medical bills across the spectrum of diabetes mellitus and atherosclerotic cardiovascular disease among non-elderly adults in the United States. J Am Heart Assoc.

[19] Caraballo C, Valero-Elizondo J, Khera R (2020). Burden and consequences of financial hardship from medical bills among nonelderly adults with diabetes mellitus in the United States. Circ Cardiovasc Qual Outcomes.

[20] Aikens JE, Piette JD (2013). Longitudinal association between medication adherence and glycaemic control in Type 2 diabetes. Diabet Med J Br Diabet Assoc.

[21] Adu MD, Malabu UH, Malau-Aduli AEO, Malau-Aduli BS (2019). Enablers and barriers to effective diabetes self-management: A multi-national investigation. PLoS ONE.

[22] Bommer C, Heesemann E, Sagalova V (2017). The global economic burden of diabetes in adults aged 20–79 years: a cost-of-illness study. Lancet Diabetes Endocrinol.

[23] Croke A, Cardwell K, Clyne B, Moriarty F, McCullagh L, Smith SM (2023). The effectiveness and cost of integrating pharmacists within general practice to optimize prescribing and health outcomes in primary care patients with polypharmacy: a systematic review. BMC Prim Care.

[24] Hincapie AL, Gupta V, Brown SA, Metzger AH (2019). Exploring perceived barriers to medication adherence and the use of mobile technology in underserved patients with chronic conditions. J Pharm Pract.

[25] Dalton K, Byrne S (2017). Role of the pharmacist in reducing healthcare costs: current insights. Integr Pharm Res Pract.

[26] Aguiar PM, da Silva CHP, Chiann C, Dórea EL, Lyra DP, Storpirtis S (2018). Pharmacist-physician collaborative care model for patients with uncontrolled type 2 diabetes in Brazil: results from a randomized controlled trial. J Eval Clin Pract.

[27] Matzke GR, Moczygemba LR, Williams KJ, Czar MJ, Lee WT (2018). Impact of a pharmacist-physician collaborative care model on patient outcomes and health services utilization. Am J Health-Syst Pharm AJHP Off J Am Soc Health-Syst Pharm.

[28] Kieu TTM, Trinh HN, Pham HTK, Nguyen TB, Ng JYS (2020). Direct non-medical and indirect costs of diabetes and its associated complications in Vietnam: an estimation using national health insurance claims from a cross-sectional survey. BMJ Open.

[29] Hird TR, Zomer E, Owen A (2019). The impact of diabetes on productivity in China. Diabetologia.

[30] American Diabetes Association (2019). American Diabetes Association. Standards of medical care in diabetes—2019. Diabetes Care.

[31] Egunsola O, Li JW, Mastikhina L, Akeju O, Dowsett LE, Clement F (2022). A qualitative systematic review of facilitators of and barriers to community pharmacists-led anticoagulation management service. Ann Pharmacother.

